# Physical activity and sleep quality among pregnant women during the first and second trimesters are associated with mental health and adverse pregnancy outcomes

**DOI:** 10.1186/s12905-024-03126-8

**Published:** 2024-08-13

**Authors:** Bin Song, Dan Wang, Xiaoli Yan, Ping Yan, Heying Liu, Hongyu Li, Shuhua Yi

**Affiliations:** Department of Gynecology and Obstetrics, First Affiliated Hospital (Army Medical University), No. 30, Gaotanyan Street, Shapingba District, Chongqing, 400038 China

**Keywords:** Physical activity, Sleep quality, Mental health, First trimester, Second trimester, Correlation analysis, Survival analysis, Prognosis

## Abstract

**Background:**

Appropriate physical activity (PA) and good sleep are beneficial to maternal and fetal health. This paper sought to explore the associations of PA and sleep quality among healthy women at the first and second trimesters of pregnancy on mental health and pregnancy outcomes.

**Methods:**

Totally 268 healthy pregnant women were retrospectively analyzed as study subjects, 134 each in the first trimester (FT) and second trimester (ST). Their baseline clinical data were obtained respectively at two stages of pregnancy. The PA/sleep quality of subjects were assessed through the Pregnancy Physical Activity Questionnaire-Chinese version (PPAQ-C)/Pittsburgh Sleep Quality Index (PSQI) scale. The mental health was assessed via the Hospital Anxiety and Depression Scale (HADS). The correlations of PA and sleep quality with mental health were analyzed using Spearman correlation analysis. Pregnancy outcomes of all subjects, associations of moderate intensity (MI) PA and sleep quality with adverse pregnancy outcomes, and independent influencing factors for adverse outcomes were analyzed.

**Results:**

Pregnant women in the ST group exhibited higher levels of MI, worse sleep quality, and lower levels of anxiety and depression than those in the FT group. Anxiety and depression were negatively correlated with MI but positively linked with PSQI scores at the first and second trimesters. MI ≥ 7.5 MET-h/week and good sleep quality were associated with a reduced incidence of adverse pregnancy outcomes.

**Conclusion:**

MI ≥ 7.5 MET-h/week and good sleep quality at the first and second trimesters of pregnancy benefit mental health and markedly reduce the occurrence of adverse pregnancy outcomes.

**Supplementary Information:**

The online version contains supplementary material available at 10.1186/s12905-024-03126-8.

## Background

Pregnancy brings women into a new stage in their lives, which involves physical and psychological alternations as well as a dynamic renegotiation of identity in the family and society [1, 2]. Women are particularly susceptible to developing various types and degrees of mental health problems during pregnancy, with depression, stress, and anxiety being the most prevalent and often occurring simultaneously [3]. The prevalence of prenatal depression is reported to range between 7 and 37.1%, and the depression is intimately related to adverse maternal and fetal health consequences [4]. Likewise, antenatal anxiety, with a global prevalence of 15-20% and higher rates reported in low- and middle-income countries, appears to be linked with preterm birth and decreased rates of breastfeeding [5]. Considering the association of altered maternal mental conditions with adverse outcomes, caring for mental health of pregnant women emerges as a high priority [6].

Physical activity (PA) refers to any body activity where movement of skeletal muscles results in energy expenditure, and it embodies a series of activity types including sports, leisure, and family and professional activities [7]. Besides, the World Health Organization updated guidelines for sedentary behavior and physical activity in 2020, prompting pregnant women to participate in moderate intensity (MI) PA (150 min/week) in household chores and daily life [8], which is related to multiple health benefits, and these benefits can be illustrated by changes in cardiovascular and metabolic processes [9]. Furthermore, it is interesting to note that MI activity contributes to conspicuously ameliorating the physical condition of pregnant women and optimizing sleep patterns, mood and health, thus preventing pregnancy-associated complications, and improving work capacity [10–12]. In light of the above evidence, appropriate PA throughout pregnancy is safe, desirable, and beneficial, and pregnant women are encouraged to engage in safe PA.

Women’s sleep is frequently disturbed during pregnancy, and nearly 80% of pregnant women experience sleep disturbances [13]. According to recent meta-analyses, insomnia, restless leg syndrome, and obstructive sleep apnea affect 38.2%, 20%, and 15% of pregnant women, respectively [14]. Essentially, pregnancy-specific changes in hormone secretion, cardiovascular, and respiration functions, fetal movement, and frequent nighttime awakenings to urinate resulting from an enlarged uterus are all contributors to poor sleep quality in pregnant women, which further elevates the risk of various maternal complications and unfavorable fetal outcomes [15]. There is evidence to suggest that MI activityis a potential and valuable adjunct treatment to combat sleep disorders during pregnancy [16–18]. Preceding researchers have also highlighted the importance of improving sleep quality for mental health [19]. A variety of previous evidence supports that MI activity is beneficial to the health of both newborn and maternity [20, 21]. For instance, regular PA during pregnancy has a bearing on reduced risks of diabetes mellitus, weight gain, preterm labor, cesarean delivery and hypertension [22–24]. Moreover, pregnancy stands out as a special stage in a woman’s life, whose health status affects both mother and child; David Barker put forward the hypothesis of “Fetal Origins of Adult Disease” in the 1990s [25, 26]; beyond that, Development Origins of Health and Disease (DOHaD) is formally proposed internationally in 2000, suggesting that growth and development in the infancy and fetal period closely links to health in adulthood, with intergenerational effects [27]. As a consequence, PA during pregnancy, as a controllable factor for adverse pregnancy outcomes, has attracted attention from scholars in public health, obstetrics and gynecology, sports medicine, demography, diet and nutrition from more and more countries and regions, and has become one of the hotspots of cross-disciplinary research. In the light of this, we hypothesized that MI activity in healthy women during first trimester (FT) and second trimester (ST) could effectively improve sleep quality, which in turn promoted the development of psychological health and reduced the occurrence of adverse pregnancy outcomes. In this context, this current study aimed to investigate the associations of PA and sleep quality at two stages of pregnancy-the first and second trimesters- with mental health and subsequent pregnancy outcomes.

## Methods

### Ethics statement

This study was conducted following the ethical principles of the World Medical Association Declaration of Helsinki and the relevant norms and regulations for clinical research. The study was approved by the academic ethics committee of First Affiliated Hospital (Army Medical University).

### Study subjects

A total of 700 healthy pregnant women who had regular prenatal care in First Affiliated Hospital (Army Medical University) from September 2021 to December 2021 were retrospectively analyzed. Based on the inclusion and exclusion criteria, 268 pregnant women were finally included in this study after excluding those who had incomplete data. They were then assigned into the FT group (*N* = 134) and the ST group (*N* = 134) according to their length of pregnancy. Baseline clinical data such as age, body mass index (BMI), gravidity, systolic blood pressure (SBP), diastolic blood pressure (DBP), fasting blood glucose (FBG), and glycosylated hemoglobin (HbA1c) were acquired respectively for all subjects at the first and second trimesters of pregnancy.

### Inclusion and exclusion criteria

Inclusion criteria: singleton pregnancy; first or second trimester of pregnancy; no pregnancy complications; complete data.

Exclusion criteria: multiple pregnancies; late trimester of pregnancy; with other diseases such as heart disease, hypertension, and diabetes mellitus; incomplete data.

The first trimester of pregnancy was defined as the period from the first week to the end of the 13th week of pregnancy; second trimester indicated the period from the 14th week to the end of the 27th week of pregnancy; late trimester indicated the period from the 28th week of pregnancy to the end of delivery.

### Clinical data collection

We retrieved the following information of all study subjects from the electronic medical record system of First Affiliated Hospital (Army Medical University), such as age, height, weight, gravidity, education level, blood pressure and blood glucose, and collected clinical data including the overall amount of physical activity, the intensity and type of physical activity, and its corresponding MET-h/week and sleep quality at the first and second trimesters of pregnancy in all subjects. At the same time, the mental health status of all subjects in mid-pregnancy was counted, with pregnancy outcomes recorded. The adverse pregnancy outcomes were defined as: premature rupture of membranes, premature delivery, postpartum hemorrhage, and cesarean delivery in pregnant women as well as low birth weight, fetal growth restriction, fetal distress, and neonatal asphyxia [28, 29].

### Assessment of PA

The Pregnancy Physical Activity Questionnaire-Chinese version (PPAQ-C) was adopted by pregnant women to self-estimate the PA in the current trimester [30]. The questionnaire can determine the PA intensity (sedentary, light, moderate, or vigorous), and may establish a pattern of PA during pregnancy and compare between various study subjects. The respondents accordingly adopted the category that could best estimate the amount of time spent on a particular activity per day or week. The time spent on each activity in the questionnaire was subsequently multiplied by its intensity. The intensity and time predictor for each question were determined following PPAQ-C instructions based on the widely-used PA compendium, thereby procuring the energy expenditure measured in metabolic equivalent of task (MET) [31]. To measure the energy expenditure per hour per week (MET-h/week), the obtained result was multiplied by the number of days in the week. According to the energy expenditure, each activity was graded with regard to intensity: (a) sedentary intensity (< 1.5 METs), (b) light intensity (1.5– < 3.0 METs), (c) MI (3.0–6.0 METs), (d) vigorous intensity (>6.0 METs) [32]. The total PA, PA intensity, and its corresponding MET-h/week were recorded at the first (8-12 weeks) and second (24-28 weeks) trimesters of pregnancy. As suggested by the World Health Organization, all adults including pregnant women should participate in at least 150 min of MI activity per week (equivalent to 7.5-15 MET-h/week) for health benefits [33]. Therefore, we analyzed the effect of PA on pregnancy outcomes based on whether study subjects achieved 7.5 MET-h/week of MI activity in the first and second trimesters of pregnancy.

### Monitoring of sleep status

The sleep quality was evaluated using the Pittsburgh Sleep Quality Index (PSQI) [34], a well-validated and standard tool for measuring sleep quality in Chinese pregnant women [35]. The PSQI consisted of a 19-item self-rating questionnaire that could assess sleep quality during the past month. The total scores ranged from 0 to 21, with higher scores indicating a worse sleep quality. In addition, a total score of ≥ 5 indicated poor sleep quality [36]. Sleep quality of all the subjects at the first (8-12 weeks) and second (24-28 weeks) trimesters of pregnancy was recorded.

### Assessment of mental health in mid-pregnancy

The anxiety and depression in mid-pregnancy were assessed using the Chinese version of the Hospital Anxiety and Depression Scale (HADS) [37, 38]. This self-administered rating questionnaire comprised 14 items, 7 items each for anxiety and depression. Each item was scored with a 4-point Likert-type scale ranging from 0 to 3. For anxiety and depression sub-scales, the minimum and maximum scores ranged from 0 to 21. The study subjects with a total score ≥ 13 or a single score ≥ 8 were considered pathological [39]. Higher scores were indicative of severer depression/anxiety symptoms. The sleep quality of participants was all recorded.

### Statistical analysis

SPSS 21.0 statistical software (IBM Corp. Armonk, NY, USA) and GraphPad Prism 8.01 (GraphPad Software Inc., San Diego, CA, USA) were performed for statistical analysis and plotting of all data. The Kolmogorov-Smirnov test was implemented to test the normal distribution. The normally-distributed measurement data were depicted as mean ± standard deviation (SD) and examined by an independent samples *t*-test. The non-normally distributed measurement data were exhibited as the median (minimum-maximum) and examined utilizing the Mann-Whitney U test. Categorical variables were compared using Fisher’s exact test. Correlations of PA (total PA and sedentary, light, moderate or vigorous intensity) and sleep quality in the first and second trimesters of pregnancy with mid-pregnancy mental health (anxiety and depression) were analyzed by Spearman rank correlation test, and the correlation coefficient for each pair of variables was calculated. Kaplan-Meier method was utilized to analyze the associations of PA and sleep quality in the first and second trimesters of pregnancy with pregnancy outcomes in healthy women, followed by Log-rank test. Baseline clinical data including age, body mass index, gravidity, HbA1c, DBP, SBP, FBG, PSQI, and MI were included in the multifactorial Cox regression model to analyze independent influencing factors for adverse pregnancy outcomes. Statistical significance was declared when *p* < 0.05.

## Results

### Clinical baseline data

Based on the length of pregnancy, the enrolled 268 pregnant women were allocated into the FT group (*N* = 134) and ST group (*N* = 134). As indicated in Table 1, no significant differences were noted in clinical baseline characteristics such as age, gravidity, SBP, DBP, FBG, and HbA1c between the two groups (all *p* > 0.05), whereas the ST group exhibited higher BMI than the FT group (*p* < 0.05).

**Table 1:** Clinical baseline data

Clinical characteristics	FT (*N* = 134)	ST (*N* = 134)	*p* value
Age (years)	29.00 (22.00-37.00)	29.00 (23.00-35.00)	0.374
BMI (kg/m^2^)	22.70 ± 1.73	23.16 ± 1.70	**0.028**
Gravidity (times)	0.00 (0.00-1.00)	0.00 (0.00-1.00)	0.625
SBP (mmHg)	117.30 ± 8.18	118.60 ± 8.32	0.206
DBP (mmHg)	73.36 ± 7.37	74.62 ± 7.43	0.166
FBG (mmol/L)	4.17 ± 0.27	4.23 ± 0.30	0.104
HbA1c (%)	4.99 ± 0.23	5.03 ± 0.24	0.132

The normally-distributed measurement data were depicted as mean ± SD and examined by an independent samples *t*-test. The non-normally distributed measurement data were exhibited as the median (minimum-maximum) and examined by the Mann-Whitney U test

*Note*: *FT* first trimester, *ST* second trimester, *BMI* body Mass Index, *SBP* systolic blood pressure, *DBP* diastolic blood pressure, *FBG* fasting blood glucose, *HbA1c* glycosylated hemoglobin

### PA and sleep quality of subjects at enrollment

The PA and sleep quality of all enrolled subjects were assessed by the PPAQ-C and PSQI scale, and the differences between the two groups were also analyzed. As shown in Table 2, there were no evident differences in total PA, and sedentary, light, and vigorous intensity PA between the two groups (*p* > 0.05); while the ST group had higher levels of MI activity and poorer sleep quality than the FT group (*p* < 0.05).

**Table 2:** PA and sleep quality of subjects at enrollment

	FT (*N* = 134)	ST (*N* = 134)	*p* value
PA (MET-h/week)			
Total PA	179.00 (70.30-315.00)	191.10 (74.20-311.20)	0.086
Sedentary intensity	32.55 (7.80-58.70)	31.05 (5.50-54.40)	0.061
Light intensity	110.80 (20.20-260.20)	109.80 (12.10-259.60)	0.649
Moderate intensity	35.05 (2.80-117.80)	44.20 (3.00-137.20)	**0.039**
Vigorous intensity	0.00 (0.00-2.10)	0.00 (0.00-2.20)	0.702
MI ≥ 7.5 MET-h/week	102 (76.12)	104 (77.61)	0.772
MI < 7.5 MET-h/week	32 (23.88)	30 (22.39)	
PSQI scores	4.00 (1.00-8.00)	5.00 (2.00-8.00)	**0.007**

The non-normally distributed measurement data were presented as the median (minimum-maximum) and examined by the Mann-Whitney U test. Categorical variables were compared using Fisher’s exact test

*Note*: *PA* physical activity, *FT* first trimester, *ST* second trimester, *MET* metabolic equivalent of task, Sedentary intensity < 1.5 METs, Light intensity 1.5-3.0 METs, Moderate intensity 3.0-6.0 METs, Vigorous intensity > 6.0 METs, *PSQI* Pittsburgh Sleep Quality Index

### Mental health in mid-pregnancy of subjects

The mental health status in mid-pregnancy was further assessed by the HADS scale to reflect the levels of anxiety and depression. The results demonstrated that the anxiety and depression levels of pregnant women in the ST group were prominently reduced compared to those in the FT group (Table 3, *p* < 0.05).

**Table 3:** Mental health in mid-pregnancy of subjects

HADS scale	FT (*N* = 134)	ST (*N* = 134)	*p* value
Anxiety	7.00 (2.00-11.00)	6.00 (2.00-10.00)	**0.039**
Depression	5.00 (2.00-9.00)	5.00 (1.00-8.00)	**0.040**

The Chinese version of the Hospital Anxiety and Depression Scale (HADS) was used to evaluate the anxiety and depression in all participants during the ST. The non-normally distributed measurement data were expressed as the median (minimum-maximum) and examined by the Mann-Whitney U test

*Note*: *FT* first trimester, *ST* second trimester

### Correlation between PA and mid-pregnancy mental health

To clarify the relationship between PA with mid-pregnancy mental health, Spearman analysis was performed to analyze the correlation of PA with anxiety and depression. As exhibited in Table 4, the total PA, sedentary, light, and vigorous PA in both groups presented no significant differences with mid-pregnancy anxiety and depression (all* p* > 0.05), while MI activity in the FT or ST group was inversely correlated with mid-pregnancy anxiety and depression (all* p* < 0.05). The aforementioned results implied that appropriate MI activity at the first and second trimesters was beneficial to mid-pregnancy mental health.

**Table 4:** Correlation of MI with mid-pregnancy mental health

HADS scale	FT (*N* = 134)		ST (*N* = 134)	
PA (MET-h/week)	Anxiety	Depression	Anxiety	Depression
Total PA	-0.129	-0.092	-0.129	-0.166
Sedentary intensity	0.002	-0.058	-0.017	0.101
Light intensity	0.036	0.069	0.063	-0.011
Moderate intensity	-0.469*	-0.378*	-0.461*	-0.432*
Vigorous intensity	-0.115	0.044	-0.101	-0.071

*Note*: *HADS* Hospital Anxiety and Depression Scale, *FT* first trimester, *ST* second trimester, *PA* physical activity, *MET* metabolic equivalent of task, *Sedentary intensity* < 1.5 METs, *Light intensity* 1.5-3.0 METs, *Moderate intensity* 3.0-6.0 METs, *Vigorous intensity* > 6.0 METs

The correlation of MI with mid-pregnancy mental health (anxiety and depression) was analyzed by the Spearman analysis. r, correlation coefficient; *p < 0.05

### Correlation between sleep quality and mid-pregnancy mental health

**Fig. 1:**
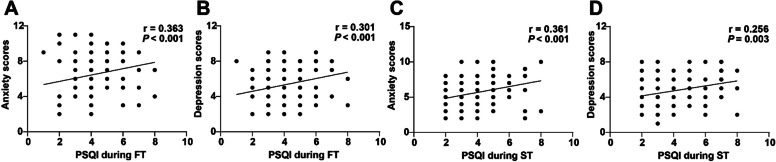
Correlation between sleep quality and mid-pregnancy mental health. Spearman analysis was used to analyze the correlation of sleep quality at first and second trimesters with mid-pregnancy mental health (anxiety and depression). **A**-**B** Correlation of sleep quality during FT with mid-pregnancy anxiety and depression; **C**-**D** Correlation of sleep quality during ST with mid-pregnancy anxiety and depression. r indicated the correlation coefficient

Subsequently, the correlation of sleep quality at the first and second trimesters with mid-pregnancy mental health (anxiety and depression) was assessed through Spearman analysis. The PSQI during FT was positively linked with mid-pregnancy anxiety (Fig. 1A, *r* = 0.363,* p* < 0.001) and depression (Fig. 1B, *r* = 0.301,* p* < 0.001); PSQI during ST was also positively related to mid-pregnancy anxiety (Fig. 1C, *r* = 0.361,* p* < 0.001) and depression (Fig. 1D, *r* = 0.256,* p* < 0.001). Since a higher PSQI score was associated with poorer sleep quality, good sleep quality at the first and second trimesters was conducive to mid-pregnancy mental health.

### Associations of PA and sleep quality with the occurrence of adverse pregnancy outcomes

**Fig. 2:**
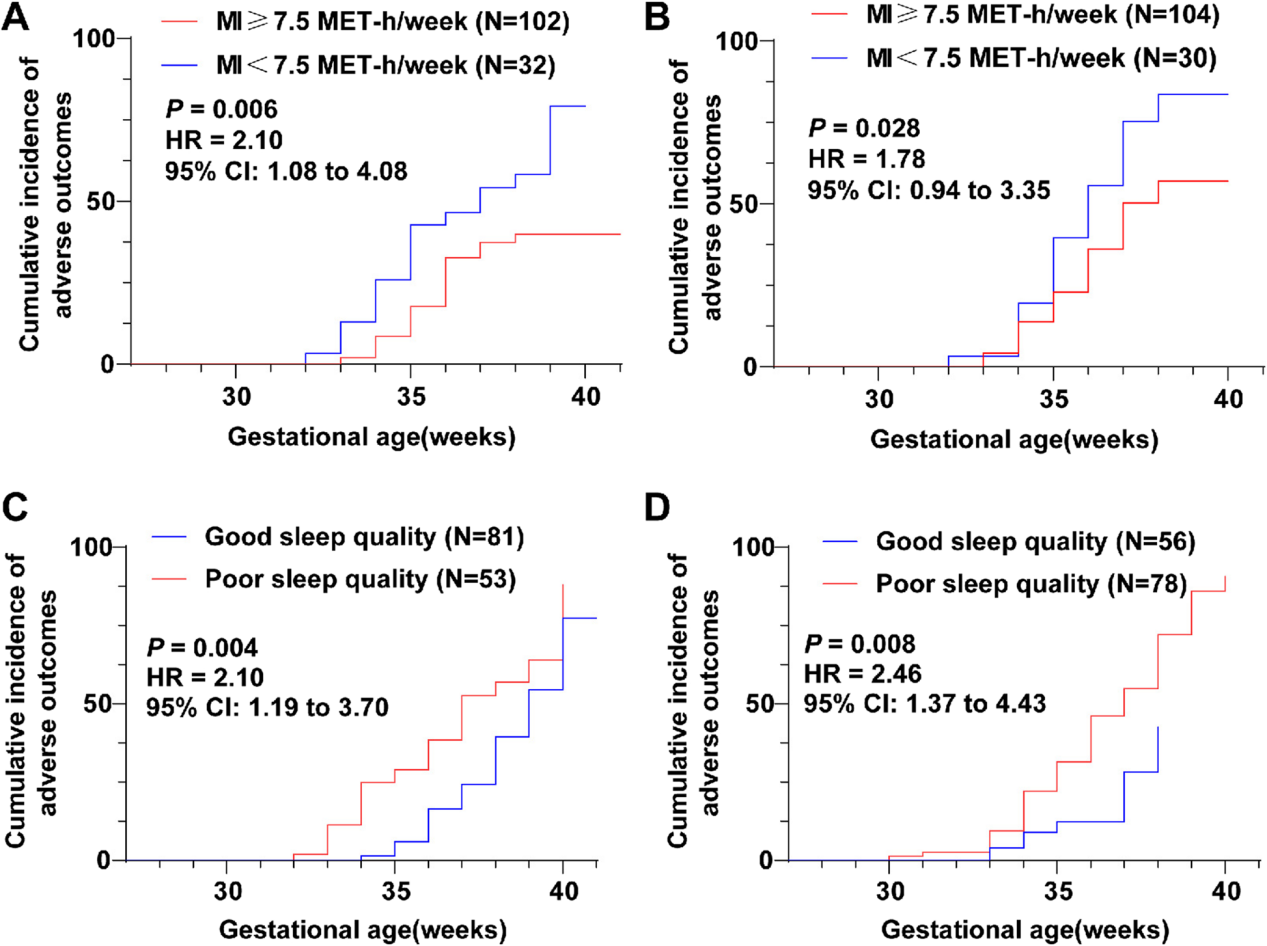
Associations of PA and sleep quality with the occurrence of adverse pregnancy outcomes. Kaplan-Meier method was employed to analyze the associations of PA and sleep quality at first and second trimesters with pregnancy outcomes among healthy pregnant women. **A** The curve of the MI < 7.5 MET-h/week group during FT was shifted to the left compared with the MI ≥ 7.5 MET-h/week group; **B** The curve of the MI < 7.5 MET-h/week group during ST was shifted to the left in comparison with the MI ≥ 7.5 MET-h/week group; **C** The curve of the poor sleep quality group during FT was shifted to the left compared with the good sleep quality group; **D** The curve of the poor sleep quality group during ST was shifted to the left compared with the good sleep quality group

According to whether the MI activity reached 7.5 MET-h/week, pregnant women in the FT and ST groups were further divided into the MI ≥ 7.5 MET-h/week and MI < 7.5 MET-h/week groups, and the incidence of adverse pregnancy outcomes was compared. The results demonstrated that the incidence of adverse outcomes was higher in pregnant women with MI < 7.5 MET-h/week during FT or ST than in those with MI ≥ 7.5 MET-h/week, respectively (Table 5, all* p* < 0.01). Kaplan-Meier analysis revealed that the curves of the MI < 7.5 MET-h/week groups during FT or ST were shifted to the left compared to the MI ≥ 7.5 MET-h/week groups (Fig. 2A-B, all* p* < 0.05), indicating that the cumulative incidence of adverse pregnancy outcomes was higher in the MI < 7.5 MET-h/week group at the same gestational week. In summary, MI ≥ 7.5 MET-h/week during FT or ST helped reduce the occurrence of adverse pregnancy outcomes.

**Table 5:** Delivery outcomes of pregnant women with different PA and sleep quality at first and second trimesters

	FT (*N* = 134)				ST (*N* = 134)			
	total	Adverse outcomes	Normal delivery	*p*	total	Adverse outcomes	Normal delivery	*p*
MI ≥ 7.5 MET-h/week	102	31	71	**0.006**	104	33	71	**0.003**
MI < 7.5 MET-h/week	32	19	13		30	19	11	
Good sleep quality	81	21	60	**< 0.001**	56	8	48	**< 0.001**
Poor sleep quality	53	29	24		78	44	34	

Categorical variables were compared using Fisher’s exact test

*Note*: *A* physical activity, *MI* moderate intensity, *FT* first trimester, *ST* second trimester, *MET* metabolic equivalent of task, Good sleep quality, Pittsburgh Sleep Quality Index (PSQI) < 5, Poor sleep quality PSQI ≥ 5

Additionally, pregnant women in the FT and ST groups were divided into the good sleep quality group (PSQI < 5) and poor sleep quality group (PSQI ≥ 5), respectively. The poor sleep quality groups had higher incidences of adverse pregnancy outcomes than the good sleep quality groups during FT or ST (Table 5, all* p* < 0.05). As indicated in Kaplan-Meier analysis, compared to the good sleep quality groups, the curves of the poor sleep quality groups during FT or ST were shifted leftward (Fig. 2C-D, all *p* < 0.05), suggesting that the cumulative incidence of adverse outcomes was higher in the poor sleep quality group at the same gestational week. Together, the above findings implied that maintaining good sleep quality during the first and second trimesters could lower the incidence of adverse pregnancy outcomes.

### Multifactorial COX regression analysis of factors affecting adverse pregnancy outcomes in healthy women

To further assess the relations of PA and sleep quality with pregnancy outcomes during FT and ST, we included age, body mass index, gravidity, SBP, DBP, FBG, HbA1c, PSQI and MI in the multifactorial Cox regression model. The results emanated that PA and sleep quality during FT and ST were independent influencing factors for adverse pregnancy outcomes in healthy women (all *P* < 0.05, Table 6-Table 7).

**Table 6:** Multifactorial COX regression analysis of factors influencing adverse pregnancy outcomes in healthy women during FT

Variable	Multivariable	
	HR (95% CI)	*P*
Age	1.024 (0.897~1.168)	0.729
BMI	0.860 (0.404~1.830)	0.696
Gravidity	0.967 (0.344~2.718)	0.949
SBP	0.846 (0.616~1.164)	0.305
DBP	1.268 (0.879~1.829)	0.205
FBG	0.278 (0.034~2.267)	0.232
HbA1c	2.999 (0.338~26.648)	0.324
PSQI during FT	1.237 (1.008~1.519)	0.042
MI activity during FT	0.988 (0.976~1.000)	0.043

**Table 7:** Multifactorial COX regression analysis of factors affecting adverse pregnancy outcomes in healthy women at FT

Variable	Multivariable	
	HR (95% CI)	*P*
Age	0.904 (0.787~1.039)	0.156
BMI	0.959 (0.674~1.366)	0.817
Gravidity	0.958 (0.398~2.302)	0.923
SBP	0.958 (0.832~1.103)	0.547
DBP	0.999 (0.917~1.088)	0.983
FBG	1.249 (0.043~36.366)	0.897
HbA1c	4.653 (0.589~36.752)	0.145
PSQI during ST	1.255 (1.001~1.573)	0.049
MI activity during ST	0.989 (0.979~0.999)	0.028

## Discussion

Our findings demonstrated that MI activity and good sleep quality during the first and second trimesters of pregnancy were both beneficial to improving mental health and lowering the occurrence rate of unfavorable pregnancy consequences. Additionally, in healthy women, MI activity during the first and second trimesters had a remarkable negative correlation with mid-pregnancy depression and anxiety, while PSQI score during the first and second trimesters had a noticeable positive correlation with mid-pregnancy depression and anxiety. MI ≥ 7.5 MET-h/week and maintaining good sleep quality brought about a prominent abation in the occurrence of adverse pregnancy outcomes during the first and second trimesters.

Firstly, as indicated by assessment results of PPAQ-C, PSQI, and HADS, pregnant women during ST presented with a higher level of MI activity and worse sleep quality, as well as lower degrees of anxiety and depression than those during FT. Much in line with our findings, mean moderate to vigorous PA varies by different trimesters: 11.5 minutes/day at FT, 14.3 minutes/day at ST, and 7.6 minutes/day at the third trimester [40]. Sleep disturbance affects more than one-half of all pregnancies and increases as gestation progresses [41]. During FT of pregnancy, women typically sleep longer and have increased daytime sleepiness; during ST, the total sleep time decreases; and at the end of the third trimester, an increase in oxytocin responsible for uterine contractions influences sleep fragmentation [42]. The prevalence rates are 22.57, 17.41, and 21.04% for anxiety and 35.64, 24.23, and 26.24% for depression at the 1st, 2nd, and 3rd trimesters, respectively [43]. Our results and previous compelling evidence underscored that MI activity, sleep, and mental health differed across pregnancy trimesters.

It is noteworthy that PA during pregnancy possesses the ability to decrease fatigue, anxiety, stress, and depression, as well as improve well-being [44]. PA can decrease depressive symptoms through numerous biological mechanisms, for instance by elevating beta-endorphins levels that are tightly linked with euphoria, enthusiasm, and improved mood and raising levels of brain neurotransmitters related to feelings of euphoria and satisfaction [45]. Furthermore, PA during pregnancy has been found to be effective in lowering the risk of perinatal depression and keeping pregnant women physically and mentally healthy [46]. Petrovic et al. have revealed that even regular walking (light intensity PA) during pregnancy obviously mitigates the symptoms of depression in pregnant women [47]. Additionally, Shakeel et al. have determined the relationship between PA and mental health during pregnancy objectively, with the results eliciting that relative to pregnant women who are inactive, pregnant women conforming to PA recommendation (> 150 MVPA min/week) have lower levels of depressive symptoms [48]. Generally, our study was consistent with the results of these previous studies.

On a separate note, good sleep quality is quite essential for physical fitness during pregnancy as well as a depressive symptoms-free mind [49]. Herein, we subsequently estimated the relationship between sleep quality with mid-pregnancy mental health. Our findings manifested that PSQI score in the first and second trimesters were dramatically positively correlated with anxiety and depression in mid-pregnancy, indicating that maintaining good sleep quality at the first and second trimesters brought about noticeable reductions in unhealthy emotions such as anxiety and depression in pregnant women. Compared to those with good sleep quality, women with poor sleep quality present with higher odds of symptoms of posttraumatic stress disorder, generalized anxiety, and antepartum depression [50]. Previous laboratory studies have found that both high-intensity and low-intensity PA elicit an enhancement in the latency of rapid eye movement and an abatement in rapid eye movement sleep [51, 52]. There is evidence supporting that sleep can be affected by PA [53], which suggests that sleep and PA may produce synergistic effects on mental and physical health. Taken together, appropriate MI activity and good sleep quality at the first and second trimesters were beneficial to mid-pregnancy mental health.

Regular PA helps the maternity restrain pregnancy weight gain, ameliorate cardiovascular function, diminish incidence of lower limb oedema and muscle cramps, promote mood stability, reduce musculoskeletal discomfort, and alleviate gestational hypertension and gestational diabetes, and it helps the fetus facilitate neurobehavioural maturation, enhance stress tolerance, and lower fat mass [54]. MI activity can increase placental functional capacity and blood perfusion, improve the supply of nutrients for fetal development, and substantially prevent preeclampsia [55]. Disturbed sleep is emerging as a prominent risk factor for adverse pregnancy outcomes [56]. Of note, obstructive sleep apnea is linked with adverse outcomes, such as gestational diabetes, maternal hypertension, preterm birth, and low infant Apgar scores [14]. Our findings further and specifically demonstrated that MI ≥ 7.5 MET-h/week and fine sleep quality contributed to decreased incidence of adverse outcomes. Moreover, a study has found lowered risks of gestational diabetes mellitus, preeclampsia and preterm birth, and mitigated mental health among women with regularly PA [57]. Yang et al. have uncovered that poor sleep quality is obiously related to enhanced rates of obstetric complications, maternal mood disturbances, and fetal adverse outcomes [58]. We conducted the multifactorial COX regression analysis and unveiled that PA and sleep quality in healthy women at first and second trimesters were independent influencing factors for adverse pregnancy outcomes.

The strength of the study highlighted that MI ≥ 7.5 MET-h/week and satisfactory sleep quality at first and second trimesters of pregnancy were both conducive to improving mid-pregnancy mental health and decreasing the occurrence of adverse outcomes. The findings provide a theoretical basis for prenatal care. Nevertheless, there are several limitations inevitably: firstly, the number of included cases and events is small; secondly, the assessment results obtained from the self-reported PPAQ-C and PSQI scale are somewhat subjective; thirdly, the current findings cannot account for the associations of PA and sleep quality with pregnancy outcomes throughout pregnancy due to the lack of data from late pregnancy. In the future, expansion of the sample size and conduction of multi-center studies are warranted to increase the credibility of results. We shall further validate the findings by assessing sleep quality and PA through objective methods such as sleep monitoring and actigraphy [56] and investigate the impacts of PA and sleep quality during late pregnancy on pregnancy outcomes.

### Supplementary Information

Supplementary Material 1.

## Data Availability

The data that support the findings of this study are available from the corresponding author upon reasonable request.

## Abbreviations

PA: Physical activity
MI: Moderate intensity
FT: First trimester
ST: Second trimester
PPAQ-C: Pregnancy Physical Activity Questionnaire-Chinese version
PSQI: Pittsburgh Sleep Quality Index
HADS: Hospital Anxiety and Depression Scale
MET: Metabolic equivalent of task
BMI: Body mass index
SBP: Systolic blood pressure
DBP: Diastolic blood pressure
FBG: Fasting blood glucose
HbA1c: Glycosylated hemoglobin
SD: Standard deviation
