# Supplementary material for: Physical activity and sleep quality among pregnant women during the first and second trimesters are associated with mental health and adverse pregnancy outcomes
Source: BMC Womens Health. 2024 Aug 13;24:455. doi: 10.1186/s12905-024-03126-8 (PMC11321155; doi:10.1186/s12905-024-03126-8)
Supplement: Supplementary file 1 — Supplementary Material 1. [file 12905_2024_3126_MOESM1_ESM.docx]

**Highlights**

1. Women at second trimester show higher MI activity and worse sleep quality.
2. Women at second trimester present lower levels of anxiety and depression.
3. MI activity is negatively correlated with anxiety and depression.
4. PSQI score is positively associated with anxiety and depression.
5. MI ≥ 7.5 MET-h/week and good sleep quality reduce incidence of adverse outcomes.

6. MI activity/sleep quality are independent influencing factors for adverse outcomes.
